# If we build it, will they come? Perspectives on pharmacy-based naloxone among family and friends of people who use opioids: a mixed methods study

**DOI:** 10.1186/s12889-022-13078-z

**Published:** 2022-04-13

**Authors:** Susannah Slocum, Jenny E. Ozga, Rebecca Joyce, Alexander Y. Walley, Robin A. Pollini

**Affiliations:** 1 Department of Behavioral Medicine & Psychiatry, School of Medicine, West Virginia University, Morgantown, WV USA; 2 Department of Community Health Sciences, School of Public Health, Boston University, Boston, MA USA; 3 Grayken Center for Addiction, Clinical Addiction Research Education Unit, Boston Medical Center, Boston University School of Medicine, Boston, MA USA; 4 Department of Epidemiology and Biostatistics, School of Public Health, West Virginia University, Morgantown, WV USA

**Keywords:** Naloxone, Pharmacies, Standing order, Opioid overdose

## Abstract

**Background:**

Expanding access to the opioid antagonist naloxone to reduce overdose mortality is a public health priority in the United States. Naloxone standing orders (NSOs) have been established in many states to increase naloxone dispensing at pharmacies, but increased pharmacy access does not ensure optimal uptake among those likely to witness an overdose. In a prior statewide purchase trial, we documented high levels of naloxone access at Massachusetts pharmacies under a statewide NSO. In this study, we characterize barriers to pharmacy-based naloxone uptake among potential opioid overdose “bystanders” (friends or family of people who use opioids) that may be amenable to intervention.

**Methods:**

Eligible bystanders were Massachusetts residents ≥ 18 years of age, did not use illicit opioids in the past 30 days, and knew someone who currently uses illicit opioids. We used a sequential mixed methods approach, in which a series of semi-structured qualitative interviews (*N* = 22) were conducted April-July 2018, to inform the development of a subsequent quantitative survey (*N* = 260), conducted February-July 2020.

**Results:**

Most survey participants (77%) reported ever obtaining naloxone but few (21%) attempted to purchase it at a pharmacy. Qualitative participants revealed that barriers to utilizing the NSO included low perceived risk of overdose, which was rooted in misconceptions regarding the risks of prescription opioid misuse, denial about their loved one’s drug use, and drug use stereotypes; inaccurate beliefs about the impact of naloxone on riskier opioid use; and concerns regarding anticipated stigma and confidentiality. Many participants had engaged in mutual support groups, which served as a source of free naloxone for half (50%) of those who had ever obtained naloxone.

**Conclusions:**

Despite high levels of pharmacy naloxone access in Massachusetts, few bystanders in our study had attempted to obtain naloxone under the NSO. Low perceived risk of overdose, misinformation, stigma, and confidentiality were important barriers to pharmacy naloxone uptake, all of which are amenable to intervention. Support groups provided a setting for addressing stigma and misinformation and provided a discreet and comfortable setting for naloxone access. Where these groups do not exist and for bystanders who do not participate in such groups, pharmacies are well-positioned to fill gaps in naloxone availability.

## Background

Deaths from drug overdose have reached historic levels in the United States, with provisional data estimating more than 93,000 overdose deaths in 2020, a 29% increase over the prior year [1]. In 2019, the most recent drug-specific data available, 71% of U.S. overdose deaths involved at least one opioid; these included synthetic opioids other than methadone (primarily fentanyl; 73%), heroin (28%), and/or prescription opioids (28%) [2]. Interventions that reduce opioid overdose risk, and accompanying interventions to improve emergency response when these overdoses occur, are critical components of ongoing efforts to reduce overdose mortality.

Expanding access to the opioid antagonist naloxone is a safe and cost-effective method to reduce opioid overdose deaths [3], and there is evidence that expanding naloxone distribution significantly reduces community-level overdose mortality rates [4, 5]. National data from the US also indicate that higher rates of naloxone access and distribution could avert between 14–21% of opioid overdose deaths [6, 7]. In April 2018, the US Surgeon General recommended that anyone who is prescribed high-dose opioids; misuses prescription opioids; uses illicit opioids; is a family member or friend of someone with an opioid use disorder (OUD); or is a community member likely to come into contact with someone at risk of opioid overdose should obtain naloxone [8]. While naloxone distribution should be prioritized for people who use illicit opioids, as they are most likely to witness and respond to overdoses [9], those who do not use opioids but may witness an overdose constitute an important target population for naloxone distribution as well. Furthermore, educating the non-using network of people who use drugs about overdose response may help reduce stigma and improve societal overdose response.

Historically, naloxone’s status as a prescription medication in the US has served as a barrier to its widespread distribution [10, 11]. To overcome this barrier, all 50 states and the District of Columbia now allow naloxone dispensing at pharmacies via a naloxone standing order (NSO) or similar mechanism [12]. These mechanisms allow pharmacists to dispense naloxone without a patient-presented prescription. Although there is evidence that NSOs significantly increase naloxone access [13–15], pharmacy participation in these programs appears to vary widely, with availability ranging from 21–93% at pharmacies across states and localities including Alabama, Georgia, Massachusetts, North Carolina, Tennessee, Texas, and Philadelphia [16–24]. Reasons for such variability include initial NSO implementation challenges, inconsistent stocking, lack of knowledge or clarity regarding NSO requirements and procedures, billing issues, and pharmacist attitudes and beliefs regarding opioid use and naloxone dispensing [11].

Massachusetts has demonstrated high rates of pharmacy-based naloxone access; a statewide phone survey showed that 90% of pharmacies had naloxone in stock [20], and a statewide purchase trial conducted by our study team successfully completed naloxone purchases at 81% of retail pharmacies, documenting high levels of both stocking and dispensing [16]. However, high accessibility does not necessarily mean people will use the pharmacy as an access point. There are multi-dimensional barriers to NSO utilization that contribute to inadequate naloxone uptake. For example, even where pharmacies regularly stock and are prepared to dispense naloxone, stigma related to drug use and addiction may interfere with purchasers’ willingness to use pharmacies as a naloxone access point [25, 26]. Lack of knowledge regarding availability of naloxone at pharmacies and naloxone costs have also been reported by potential naloxone consumers as barriers to pharmacy-based uptake [27].

Massachusetts provides an optimal setting for exploring whether persons at high likelihood of witnessing an overdose will utilize pharmacies as a source of naloxone where a NSO has been effectively implemented. Massachusetts has been a leader in both community-led and state-sponsored efforts to expand naloxone access, beginning in 2007 with a statewide overdose education and naloxone distribution program that ramped up naloxone distribution through syringe services programs (SSPs), support groups, and other community programs. A subsequent study demonstrated that the state’s overdose education and naloxone distribution program was associated with reductions in overdose mortality [5]. Nonetheless, retail pharmacies are well-positioned to fill continuing gaps in naloxone coverage, particularly among persons who do not use illicit drugs and therefore may not avail themselves of SSP-based naloxone distribution and those who are not able or willing to utilize other naloxone distribution points.

In addition, although existing US research has explored perspectives on naloxone from pharmacists and pharmacy staff [21, 28–36], other healthcare providers [37–41], law enforcement [42–48], and the general public [49–51], there remains a gap in knowledge regarding the perspectives of family members and friends of people who use illicit opioids (particularly those who do not currently use illicit opioids themselves), despite US Surgeon General guidelines [8], and the high likelihood that they will be called upon to respond to an overdose [52]. One study reported that as many as 35% of parents of children who use illicit opioids (PWUIO) have been witness to an opioid overdose [53]. In another study, the majority of “family carers,” most of whom were parents of a child using heroin, wanted overdose response and naloxone training [54]. In this study, we examine the willingness of potential opioid overdose “bystanders” (defined in this study as individuals who did not use illicit opioids in the past 30 days but have a close relationship with someone who does) to obtain naloxone under the Massachusetts NSO program and characterize specific knowledge, attitudes, and beliefs that may serve as barriers to pharmacy based naloxone uptake.

## Methods

This study is part of the second phase of a multi-phase, sequential mixed methods study [55, 56] designed to examine implementation and utilization of the Massachusetts NSO from the perspective of PWUIO, other potential bystanders, and pharmacists. The study employed both quantitative and qualitative methods and each phase informed the subsequent phase. In Phase I, we conducted a statewide naloxone purchase trial that found naloxone stocking and dispensing to be very high at retail pharmacies [16]. We then used a pharmacist survey and pharmacist focus groups to assess barriers and facilitators to NSO implementation [31]. In Phase II, we used semi-structured qualitative interviews and a subsequent survey to examine barriers and facilitators to utilizing the NSO from the perspective of potential overdose bystanders. Results from the Phase II qualitative and quantitative study phases were triangulated and mixed methods results are reported here.

### Setting

In 2014, the Massachusetts Department of Public Health expanded its efforts beyond the aforementioned overdose education and naloxone distribution program by empowering pharmacies to voluntarily obtain a standing order to dispense “naloxone rescue kits” consisting of two naloxone doses and a standardized naloxone pamphlet. This was followed by a requirement that all pharmacies obtain a standing order by December 1, 2017 [57]. A statewide standing order that covered all pharmacies went into effect on October 4, 2018 [58]. The state’s standing order allows naloxone to be dispensed to “any person at risk of experiencing an opioid-related overdose, as well as…family members, friends or other persons in a position to assist individuals at risk of experiencing an opioid-related overdose.” The 2018 NSO also requires all pharmacies to maintain “a continuous, sufficient supply of naloxone rescue kits, or other approved opioid antagonist medication, to meet the needs of the community.” The law stipulates that at the time of purchase, the pharmacy must provide the purchaser with counseling and a pamphlet that includes information on how to recognize and respond to an opioid overdose and how to administer naloxone. The NSO also requires that pharmacists make a reasonable attempt to determine if the purchasers’ insurance will cover naloxone [58]. Pharmacists in Massachusetts report that insurance co-pays for naloxone vary widely [30], and our prior statewide purchase trial determined that without insurance, the median out of pocket cost for naloxone (usually single-step nasal naloxone, brand name Narcan®) in Massachusetts was $133.38 [16]. At the pharmacy counter, patients may provide their name or have “Naloxone Rescue Kit” entered in lieu of this identifiable information for purposes of the standing order, and dose numbers are reported by pharmacies to the state without patient identifiers [58].

### Bystander qualitative interviews

#### Recruitment

We partnered with a Massachusetts-based non-profit support network for parents and family members of persons with a substance use disorder (SUD) to recruit eligible participants. Recruitment efforts focused on the North and South Shore areas of Massachusetts in accordance with the parent study’s overall geographic recruitment strategy, which focused on two regions with similarly high overdose fatality rates. Study staff attended support group meetings to explain the study and invite participation, and potential participants were screened in person or by follow-up phone call. Eligibility criteria were ≥ 18 years of age, not using illicit opioids (e.g., heroin, street fentanyl, pharmaceutical opioids not prescribed for their use) in the past 30 days, having a close relationship with someone who currently uses illicit opioids, and willingness to provide informed consent. Massachusetts residency was a requirement for participation.

### Data collection

Between April and July 2018, we conducted 22 semi-structured interviews (7 South Shore region, 15 North Shore region). Interviews were conducted in locations that were mutually agreed upon by participants and research staff and included coffee shops, private homes, and a private office within a SSP. Qualitative interviews included a brief quantitative section in which participants self-reported demographics and their experience with naloxone acquisition, overdose training, and overdose response. The in-depth interview consisted of an open-ended conversation, allowing for emergence of new information. We used a topic guide to ensure coverage of broad predetermined areas of inquiry including overdose risk perceptions and experiences, naloxone knowledge, attitudes about accessing naloxone in pharmacies and other settings, and experiences with health services including how perceived and experienced stigma may influence willingness to access naloxone in different settings (e.g., “In general, how do you think people view naloxone/Narcan?”). Interviews were digitally recorded and typically lasted 60–90 min. Participants were reimbursed $40 for their time. All procedures were approved by West Virginia University’s Institutional Review Board.

### Data analysis

Interview recordings were transcribed verbatim and verified and sanitized by research staff. Participant names were replaced with pseudonyms to protect privacy. Themes were identified through a close reading of transcripts. A preliminary coding scheme was generated based on the primary domains of the interview guide (deductive) and emergent themes (inductive). A codebook was developed from the coding scheme and further emergent themes. Codes were arranged in a hierarchical structure by parent codes (e.g., naloxone) and subcodes (e.g., pharmacies, other sources). The codebook was piloted by two independent coders across six bystander interview transcripts, comparing coding for consistency and refining the codebook as required. After the codebook was finalized, the remaining transcripts were coded by the first author in consultation with the principal investigator.

For the current analysis, we examined attitudes towards naloxone as well as actual and anticipated experiences with obtaining naloxone under NSO. The authors read through the segments assigned to naloxone and segments otherwise coded that overlapped with naloxone codes; these included the deductive codes “pharmacy,” “other sources,” and “overdose” and the inductive codes “stigma,” “family dynamics,” “addiction,” and “denial.” Additional memos were written for these segments to identify cross-cutting themes and generate a deeper understanding of the data.

### Bystander quantitative surveys

#### Recruitment and data collection

Between February and July 2020, 260 bystander surveys were completed. Eligibility criteria were the same as for qualitative interviews. Survey participant recruitment began through the same non-profit support group network from which qualitative participants were recruited. To reach a broader sample of the population, bystanders also were recruited from a range of community support groups that serve friends and family of PWUIO, and by posting recruitment fliers on the social media accounts (e.g., Facebook, Instagram) of support groups based in Massachusetts. Survey questions were informed by the bystander qualitative interviews previously described and included questions on naloxone sources, access, and utilization; attitudes and beliefs toward substance use; social supports; and overdose and drug treatment navigation experiences. All questions were presented in a multiple-choice format with categorical response options. Surveys were interviewer-administered to participants over the phone and entered into REDCap software (projectredcap.org). Surveys took approximately 30 min to complete. All participants were sent $40 electronically for survey completion, either via Amazon gift card or PayPal payment.

### Data analysis

Descriptive statistics were used to summarize findings across the aforementioned question categories. Analyses were performed using R statistical software version 3.5.1 (r-project.org).

### Data triangulation

We used themes identified from interview transcripts to understand and expand upon findings from the quantitative survey regarding bystander knowledge, attitudes, and beliefs toward naloxone, as well as facilitators and barriers to naloxone access generally, and more specifically to pharmacy-based naloxone. Specific text excerpts were selected from interview transcripts to provide context for, and build upon, quantitative findings.

## Results

### Participant characteristics

Overall, demographic characteristics were comparable across bystanders who completed the qualitative interviews and quantitative survey. Qualitative participants (*N* = 22) were majority female (63%), White/Caucasian (95%), and the median age was 54 years (interquartile range (IQR) = 46 to 61 years). Survey participants (*N* = 260) were also majority female (66%), White/Caucasian (92%), and the median age was 53 years (IQR = 37 to 61 years). Most qualitative participants had one or more adult child (63%), close friend(s) (14%), sibling(s) (14%), or a spouse/partner (9%) that used illicit opioids. The median number of people qualitative participants knew who were at risk of an opioid overdose was 13 (IQR = 6 to 30). For quantitative surveys, participants had one or more adult children (34%), close friend(s) (45%), other family member(s) (17%), sibling(s) (13%), a spouse/partner (6%), and/or parent(s) (3%) that used illicit opioids. The median number of people that survey participants knew who used opioids was 10 (IQR = 4 to 30), of which a median of 2 (IQR = 1 to 6) were self-reported as “close” relationships. Most participants (67%) had ever witnessed an opioid overdose; of these, 53% had administered naloxone at least once and 65% has witnessed an opioid overdose without having naloxone on hand at least once.

### Naloxone uptake and utilization of NSO

Bystander survey outcomes are shown in Table 1. A majority of those surveyed (77%) reported that they had ever purchased or otherwise obtained naloxone. Half of participants who had ever obtained naloxone received it from a support group. A minority of survey participants obtained it from SSPs (20%) and other health services settings (20%). Fewer than half (31%) had ever obtained naloxone from a pharmacy, and even fewer (21%) reported ever attempting to purchase naloxone at a pharmacy under the NSO. However, most (68%) were aware of the NSO. Almost all qualitative participants (95%) also reported acquiring naloxone on at least one occasion, which occurred mostly through support groups (66%) and less frequently through SSPs (14%) and pharmacies (4%).

**Table 1:** Characteristics of naloxone access, source utilization, and beliefs among potential opioid overdose “bystanders” (N = 260) surveyed in Massachusetts, 2020

	**Total *N* = 260**
	***n* (%)**
Ever purchased or otherwise obtained naloxone	201 (77)
Median number of times obtained naloxone (interquartile range (IQR))	3 (2–7)
Reason(s) for not obtaining naloxone	59 (23)
Don’t need it^a^	50 (85)
Not comfortable asking for it^a^	3 (5)
Don’t know where to get it^a^	2 (3)
Confidentiality concerns^a^	2 (3)
Too expensive^a^	1 (2)
Places where naloxone has been obtained	201 (77)
Support group^b^	100 (50)
Pharmacy^b^	63 (31)
Syringe service/harm reduction program^b^	41 (20)
Hospital, physician’s office, health clinic, health department, and/or EMT^b^	41 (20)
Treatment or recovery center^b^	38 (19)
Friend/family member^b^	14 (7)
Police department^b^	4 (2)
Fire department^b^	1 (1)
Know how to use naloxone	237 (91)
Everyone in the home knows where naloxone is and how to use it^b^	114 (57)
Have recommended naloxone to others	196 (75)
Knowledge, attitudes, and beliefs about naloxone (*n* (%) who strongly agree or agree)	
Everyone who has a close family member or friend who uses illicit or prescription opioids not prescribed for them should have naloxone	258 (99)
I would be comfortable going to a syringe exchange/harm reduction program to get naloxone	217 (83)
Naloxone is just a band-aid on the drug problem	56 (22)
Everyone should carry naloxone, regardless of whether they know someone who uses opioids or not	190 (73)
Concern about stigma keeps people from getting naloxone	225 (87)
Knowing naloxone is available makes people who use drugs act more irresponsibly	64 (25)
Naloxone is affordable	145 (56)
Concern about confidentiality keeps people from getting naloxone at the pharmacy	166 (64)
I want to receive counseling every time I purchase naloxone at the pharmacy	111 (43)
I worry that people will judge me negatively if they find out I have naloxone	58 (22)
Knowing naloxone is available encourages drug use	39 (15)
I do not want to have a record of naloxone purchase on my insurance or medical record	66 (25)
Naloxone is still effective after its labeled expiration date	136 (52)
Aware that naloxone can be purchased at a pharmacy without a prescription	177 (68)
Have tried purchasing naloxone at a pharmacy without a prescription	54 (21)
Reason(s) for not trying to purchase naloxone at a pharmacy without a prescription	205 (79)
Already have a reliable free source^c^	87 (42)
Don’t need it^c^	79 (39)
Thought a prescription was needed^c^	31 (15)
Too expensive^c^	8 (4)
Worried what people will think^c^	5 (2)
Confidentiality concerns^c^	4 (2)
Not comfortable asking for it^c^	4 (2)
Don’t know where to get it^c^	4 (2)
Median number of times tried to purchase naloxone at a pharmacy without a prescription (IQR)	2 (1–4)
Have successfully purchased naloxone at a pharmacy without a prescription^d^	47 (87)
For first pharmacy purchase attempt^e^:	68 (26)
Felt comfortable going to the counter to ask for naloxone^f^	50 (74)
Expected the person at the pharmacy counter to know exactly what you were asking for^f^	60 (88)
Expected the person at the counter to treat you with respect^f^	55 (81)
Were worried what the pharmacy staff would think of you when asking for naloxone^f^	27 (40)
Were worried about confidentiality^f^	30 (44)
Got all the information needed during the purchase to confidently respond to an overdose^f^	30 (44)
Would go to a pharmacy for naloxone again^f^	54 (79)

^a^Percentages calculated based on *n* = 59 who had never purchased or otherwise obtained naloxone; ^b^Percentages calculated based on *n* = 201 who had ever purchased or otherwise obtained naloxone; ^c^Percentages calculated based on *n* = 205 who had never tried to purchase naloxone from a pharmacy without a prescription; ^d^Percentage calculated based on *n* = 54 who had ever tried to purchase naloxone at a pharmacy without a prescription; ^e^Includes pharmacy naloxone purchase attempts with and without a prescription; ^f^Percentages calculated based on *n* = 68 who had tried to purchase naloxone at a pharmacy with or without a prescription

### Barriers to naloxone uptake by bystanders

Qualitative interviews revealed barriers to accessing naloxone fell into two categories: those regarding naloxone generally and those specific to pharmacy naloxone. For general naloxone access, barriers included participants’ perceived need for and attitudes and beliefs toward naloxone. Notably, qualitative participants reported that support groups were not only an important access point for naloxone but played a critical role in dismantling psychological barriers preventing them from acquiring the medication.

### Perceived need for naloxone

Among surveyed bystanders who had never obtained naloxone (23%), a majority reported that they didn’t think they needed it because they rarely or never saw their loved one at risk of overdose. In the qualitative interviews, three related themes emerged regarding bystanders’ assessment of their loved ones’ risk of overdose and the need for naloxone: opioid formulation (specifically prescription pills versus heroin/street fentanyl), denial around the severity of their loved one’s drug use, and pre-existing stereotypes about the behavior and appearance of people at risk of overdose.

In terms of the first theme focused on opioid formulations, several participants perceived little to no overdose risk when they knew their loved one was misusing prescription pills but was not known to be using heroin/street fentanyl. They expressed that their risk underestimation was tied to their own fear of losing their loved one, for which denial became a coping mechanism. For instance, Clare (age 51), whose adult son was in recent recovery from heroin, told us that early in his drug use she did not take home a free naloxone kit offered by her support group. When asked why she had thought her son was not at risk of overdose, she said, “*I guess I didn’t think he was using. I thought he was just using Percs [Percocets],*” indicating that her risk perception was stratified by opioid formulation.

Rita (age 50), whose husband used prescription pills but was not known to use heroin, did not perceive that she would need naloxone for him:

*“I've never thought it in my head that I needed it for my husband. It was always for somebody else…I don't know why I don't think he would [overdose]. I don't know if it's because it's not heroin and it's not- you know he's not shooting up. He's not. You know maybe because in my head that's so much worse, and that's um, I don't know (laughs). I honestly don't. And then there's part of me that thinks maybe I just don't wanna- I don't wanna think about it, you know … I just kind of put it in the corner.”*

Others echoed similar accounts of denial, speaking on the ongoing process of coming to terms with the reality of drug use in their family. Maggie (age 48), whose son was in and out of recovery, was asked why she thought some people who should have naloxone didn’t have it. She recalled her own denial in an earlier stage of her son’s drug use:

*“So I guess, you have blinders on - just um, being in denial of somebody's using, which most families are I would say at the beginning of everybody's use. You know? Um, yeah. I would say if you see your kid smoking a joint, get some Narcan. You know?”*

Many qualitative participants reported that the continuation of their denial was enabled by their loved one’s efforts to conceal their drug use. For instance, Bruce (age 73) whose son was in and out of recovery for heroin/street fentanyl, spoke about his struggle with denial, *“I think they all are, uh, they're very sharp at shading the truth and not telling the truth and helping you remain in denial.”*

Pre-existing stereotypes surrounding the appearance and behavior of PWUIO additionally interfered with bystanders’ ability to pick up on signs of drug use, which inhibited their perceived need for naloxone. Katerina (age 23), whose older brother was actively using heroin, reported that early in his drug use her brother’s appearance didn’t align with her idea of people at risk of overdose: “*I didn't think of my brother as at risk [of overdose] or anything. I thought that injection drug users were, you know, homeless people living on the street. I didn't think that they were people living functional lives, with families who might have grown up in a nice place*.”

Most qualitative participants had used support groups and noted that they played an important role in accepting the severity of their loved one’s drug use and the need for naloxone. Indeed, support groups were a critical naloxone access point for many; half (50%) of the survey participants who reported ever obtaining naloxone got it from a support group. Such social support normalized bystanders’ experiences and decreased isolation and secrecy. Support groups also provided education about the neurobiology of addiction. As stigma diminished through group participation, bystanders reported that their attitudes towards naloxone were more accepting and their perceived need for the medication increased.

### Bystander attitudes and beliefs about naloxone

Most qualitative participants held positive attitudes and beliefs about naloxone as reflected in the words of Saul (age 61): “*I know many, many, many, many parents who carry it. I think it is absolute lifesaver, it's one of the best investments we can possibly make at the federal, state, and local levels.*” Similarly, most surveyed participants disagreed or strongly disagreed with the following statements derived from the prior qualitative interviews: “Naloxone is just a band-aid on the drug problem” (76%); “Knowing naloxone is available makes people who use drugs act more irresponsibly” (72%); and “Knowing naloxone is available encourages drug use” (83%). Qualitative interviews revealed that those who were unsupportive of naloxone believed it encouraged reckless drug use and discouraged recovery: *“It’s a tricky thing because you know, people they want to chase that best high they can get, and if they're using too much and know that they have Narcan to put them back, then we couldn't ever stop them from using”* (Phoebe, age 56) and *“I don't mean to sound crude but I'm just saying, if you see more of your loved ones, you know, passing away or dying because of that, wouldn't you be more susceptible of not doing it [using opioids]?”* (Rita, age 50).

Along these same lines, several qualitative participants reported that they had naloxone at home but concealed it from their loved ones, as they felt it would be construed as a “crutch” or a “backup plan.” Over half (57%) of surveyed bystanders who had ever obtained naloxone reported that everyone in the home knows where their naloxone is and how to use it. One interview participant, Audrey (age 54), recounted a situation in which her son returned to her house after overdosing the previous day and indicated to her that he wanted to use again. She worried that she was enabling him to keep using drugs:

*“. . .but I don't like … the mental thought that it's a backup plan kind of thing almost. Like, ‘Oh, just keep doing heroin. I've got my Nar- I've got Narcan.’ You know what I mean? I don't think that's a good idea. And that's what I did.”*

### Barriers to utilizing pharmacies as naloxone source

A minority (21%) of surveyed bystanders had ever tried to purchase naloxone at a pharmacy without a prescription, with the majority (87%) of those attempts resulting in a purchase. Of those who had made purchases, most (74%) reported feeling comfortable going to the counter to ask for it and 80% had expected to be treated with respect by pharmacy staff. However, 40% reported that were worried about what pharmacy staff would think of them and 44% expressed concerns about confidentiality.

For surveyed bystanders who had never tried purchasing naloxone from a pharmacy without a prescription (79%), the most common reason was that they already have a reliable, free naloxone source (42%) and thus felt no need to acquire it through pharmacies. This finding was echoed in the qualitative interviews, in which most participants reported getting free naloxone from support groups. Interview participants also spoke of a lack of knowledge surrounding the NSO, which served as a barrier to utilizing pharmacies for naloxone purchase. For instance, Delilah (age 62), whose nephew was in recovery, questioned whether every pharmacy had a standing order:

*“I don't even understand how you get it from the pharmacy... Like can I just go into a pharmacy and say, ‘I wanna purchase a Narcan?’ Or do I need to go to my physician and get a prescription? ... Like what is a standing order? … a lot of people don't even know what that is, you know? That's what I don't really understand. And is it like every pharmacy that has a standing order? So like, does every pharmacy in the city of Gloucester have a standing order? Every pharmacy in the city of Peabody, or is it just this Walgreens and this CVS that you can go to? Like-- nobody knows.”*

Given that a large proportion of participants had never attempted to obtain naloxone from a pharmacy, we asked interview and survey participants to imagine a hypothetical pharmacy purchase to learn about what they anticipated. Although most surveyed participants (90%) reported that they would feel comfortable going to the pharmacy counter to ask for naloxone, bystanders across data collections anticipated confidentiality concerns and stigma.

As an example, Katerina (age 23) spoke of not wanting to purchase naloxone in a pharmacy because of concerns about future negative consequences:

*“I don't know, like, my rights walking into the pharmacy to buy it. I don't know if they're like, ‘Who's this for?’ And then, you know, an officer shows up at my brother’s door. Like, I don't know. Like, I could see how someone would be scared that that's going to happen. Cause like, you're buying it. You're buying it because someone uses illegal drugs. It's just like, a weird thing.”*

Many others who were interviewed shared Katerina’s concerns about loss of confidentiality in the pharmacy, and 64% of those surveyed reported that they believe confidentiality concerns keep people from acquiring naloxone from pharmacies. Confidentiality concerns were often closely tied to perceived secondary stigma around having a family member or partner who used drugs. Some surveyed bystanders (35%) reported that they were worried what pharmacy staff would think of them when they ask for naloxone, and most (87%) indicated that they believe stigma concerns kept people from getting naloxone. Many interview participants also worried about possible confidentiality violations if the pharmacy staff were members of the community. For example, Maggie (age 48) spoke about her discomfort with going to her regular pharmacy for naloxone because she worried the technician would gossip about her to other community members: *“I think she [pharmacy technician] might know some of my friends, you know? So it's like, uh, is she going to go around and say, ‘I saw so-and-so's mother here buying Narcan.’* “

Edwin (age 49) who had many close friends at risk of overdose, knew his local pharmacist’s discriminatory attitude towards people who use drugs from prior encounters and did not feel that pharmacy would be a reliable resource for someone seeking naloxone:

*“I just know that there's this one pharmacy here … And the lady that runs it is a fucking asshole. And I just don't go there unless I absolutely have to. She doesn't believe in addiction, she's told me that. And, it's just I would never go in there and ask her for Narcan, 'cause I imagine she'd make it very difficult.”*

Some surveyed participants reported not wanting to have a naloxone purchase on their insurance or medical record(s) (25%) and 30% stated that they would be more likely to purchase naloxone at a pharmacy if it were offered over the counter. Several interviewed participants thought that over-the-counter naloxone would offer more discretion to customers who were embarrassed or who anticipated judgement and discrimination. Saul (age 61) explained:

*“So much of it goes back to stigma, right? People are afraid to go, you know, up and even ask. It would be much better if it was just on the shelf, that's for sure, we could just go and grab it. It’s in with all your groceries, or whatever it is that you're buying, and people aren't regu- you know, ringing it up, they're not gonna give it second, a second thought.”*

While making naloxone available over-the-counter may address concerns about medical records and patient embarrassment, it could increase out of pocket cost and would eliminate pharmacist counseling about overdose response.

## Discussion

In this study, we explored naloxone uptake among potential opioid overdose “bystanders” – a group identified by the US Surgeon General as being a priority for naloxone distribution [8] – and the factors that influence their utilization of Massachusetts’ widely implemented NSO program. Our findings revealed that some bystanders were not equipped with naloxone because they believed their loved one was at low risk of overdose; this was because their loved one did not inject, used pills rather than heroin/street fentanyl, or were not viewed as having their life destabilized by drug use. Among those equipped with naloxone, the majority had received it from support groups, not pharmacies, and their reasons included convenience and concerns about stigma and confidentiality. This work contributes to the limited literature on naloxone uptake among persons who do not use illicit opioids but have a high likelihood of witnessing an opioid overdose.

Naloxone is readily accessible at Massachusetts pharmacies to both people at risk of overdose and potential opioid overdose bystanders [16], yet many bystanders are uncomfortable due to fear of being stigmatized and have easy access to it through other more acceptable sources. Previous research indicates that bystanders who access and are trained to use naloxone have positive outcomes in terms of increased knowledge, confidence, and frequency of overdose reversals [33, 59, 60]. A majority of participants in this study reported having naloxone; however, few had obtained it from a pharmacy despite broad awareness of the NSO. Our qualitative findings suggest that the ways in which bystanders perceive others’ overdose risk may have a substantial impact on seeking naloxone, and that these perceptions are built upon a complex web of misconceptions, denial, and drug use stereotypes.

We found misconceptions in how bystanders perceive the relative risk of different opioid formulations. Bystanders reported that they initially lacked knowledge around overdose risk when their loved one was misusing prescriptions, and some reported thinking that heroin could only be administered by injection. While illicit fentanyl and heroin are involved in the majority of opioid overdose deaths [61], 30% of deaths during the period of data collection involved a prescription opioid [2, 62]. Bystander perspectives on relative risk of opioid formulations are further complicated by the evolving risk factors of the drug market, specifically the increasing prevalence of counterfeit prescription pills that often contain illicitly manufactured and potent fentanyl [63, 64]. Ultimately, opioid overdose risk assessment should focus on polysubstance use that may involve combinations of prescribed and illicit opioids, as well as benzodiazepines and other substances [65]. Providing bystanders with information that will enable them to more accurately judge their loved ones’ risk, specifically as it relates to prescription misuse, polysubstance use, and the risks of illicit supply, are essential for maximizing naloxone saturation among bystanders and their potential for overdose reversals.

In addition to these misconceptions, we found that denial was frequently mentioned as influencing bystanders’ assessment of overdose risk. Periods of denial often are experienced by people who face life altering circumstances [66–68], and learning that a loved one is living with SUD is an example of such a disruptive event [69]. Indeed, many bystanders experience a period of denial after learning of their loved one’s substance use [70]. However, denial can lead to unintended negative consequences. In this study, participants who reported an initial period of denial were largely unwilling to acquire naloxone, as they believed it was unneeded. Thus, bystanders’ denial regarding overdose risk may lead inadvertently to lack of preparedness for overdose response. In fact, more than half of participants had witnessed an opioid overdose without having naloxone on hand at least once. Based on these findings, it is possible that witnessing an opioid overdose may serve as a motivational factor for obtaining naloxone. Further exploration of whether denial interacts with misconceptions around the risk of different opioid formulations may be helpful for creating effective interventions to increase naloxone uptake among bystanders.

Our data also revealed that stereotypes about PWUIO influenced overdose risk perceptions. For some bystanders in our study, such stereotypes kept them unaware or in denial of opioid misuse when the appearance and behavior of their loved one contradicted these stereotypes, which then negatively affected naloxone acquisition. It is well established that public stigmatization of people who use drugs manifests in pervasive stereotypes and is increasingly recognized as a serious public health concern [26, 71]. In this study, bystanders also experienced secondary stigma based on their loved ones’ drug use that affected their willingness to access naloxone in a pharmacy, particularly in smaller communities where they were more likely to encounter people they knew. Further research into how family members enact drug-related stigma and how it affects their relationship with loved ones is needed to understand how stigma influences bystander response to overdoses. A better understanding of the role of stigma in this context could also contribute to more effective social messaging and supports around opioid-related risks. Classifying naloxone as an over-the-counter medication would eliminate the need for interaction and documentation at the pharmacy counter and would likely increase naloxone uptake among those with stigma and confidentiality concerns.

We also found that bystanders held misconceptions about naloxone, echoing previous research [72, 73], and that these misconceptions inhibited naloxone uptake. Specifically, some bystanders believed naloxone encourages riskier drug use and/or discourages recovery. Although this is one of the first studies to document bystander attitudes toward naloxone, prior research has documented attitudes and misconceptions about naloxone among pharmacists [21, 28–31, 33, 34, 36, 39, 74–78] and the general public [49, 51, 79]. Additionally, some medical providers are reluctant to prescribe naloxone based on the belief that treating SUD and overdose prevention are mutually exclusive [37, 40]. While beliefs about naloxone causing higher risk drug use are widespread, they are largely unfounded; research indicates that acquisition of naloxone by PWUIO does not increase high risk behaviors [80] and may actually decrease high risk drug use behaviors [81]. Such misconceptions may significantly influence support for naloxone; in contrast, better understanding of OUD is associated with greater support and acceptance of harm reduction programs, including naloxone distribution [72], indicating that interventions that increase compassionate awareness of OUD may positively impact naloxone uptake.

The majority of participants who had acquired naloxone did not do so at a pharmacy, however those who did largely had positive experiences despite worries about what pharmacy staff might think of them and negative encounters with pharmacy staff. Limited pharmacy acquisition was the result of several interconnected barriers, and partly attributable to the ease with which it was accessed through support groups. First, participants’ lacked confidence regarding how to access medications through the NSO. Second, participants experienced uncertainty and concern over future negative consequences of purchasing naloxone. Finally, embarrassment and confidentiality concerns were repeatedly cited as barriers to NSO utilization. Contrary to bystanders’ perceptions about possible future discrimination, most pharmacists are supportive of naloxone, do not hold discriminatory attitudes towards naloxone patients, and are largely concerned that offering naloxone will offend or embarrass patients [31]. Additionally, pharmacy technicians are highly willing to provide naloxone and overdose education [35]. Universal offers of naloxone may be a useful intervention to close the gap between the perceptions of bystanders and pharmacists. Indeed, naloxone consumer groups express support for universal offers of naloxone by pharmacy staff [28], and pharmacy staff report that the policy would address their own concerns about stigmatizing naloxone consumers [31].

Network connections established at mutual support groups allowed many bystanders in our study to overcome barriers to naloxone acquisition, decreasing self-deception and stigma while educating bystanders about risk assessment and harm reduction. Mutual support groups also served as the foundation for our recruitment efforts and were a primary source of naloxone and overdose training for our study participants. Many of our participants reported having free, reliable naloxone via support groups, which was a prominent factor in their low utilization of pharmacy-based naloxone. However, support groups are not universal nor are they evenly distributed across communities affected by the opioid crisis. Further, even in areas where support groups are available, the COVID-19 pandemic has forced cancellation or transition to online platforms that do not allow for naloxone distribution. Pharmacies therefore remain a key distribution point for naloxone and addressing the factors that hamper NSO utilization among potential bystanders remains important for reducing opioid overdose mortality. This study documents barriers to pharmacy naloxone uptake in an environment with high accessibility and suggests that reclassifying naloxone as an over-the-counter medication would alleviate some of these barriers.

### Limitations

Limitations for our study are largely related to the generalizability of findings. First, our sampling approach was facilitated by collaborating with local support groups, and our sample therefore reflects participation in those groups. Second, our participants largely identified as White/Caucasian, despite the fact that our targeted recruitment areas were significantly more racially and ethnically diverse and Massachusetts as a whole is 80% White/Caucasian [82]. Bystanders from racial and ethnic groups that have been historically excluded from access to high quality healthcare and harm reduction services, including Black, Latinx, and American Indian/ Alaskan Native people, likely face additional barriers to accessing naloxone. Intersectional stigma due to discrimination and racism result in further inequities in overdose prevention resources, and recent data document increases in opioid overdose mortality among non-Hispanic Black individuals [83]. Third, a majority of participants were parents to adult children with OUD and may not be generalizable to other types of “bystanders.” Fourth, Massachusetts was an early leader in implementing community- and pharmacy-based naloxone distribution programs, including community education efforts, and our findings therefore may not be generalizable to states in earlier stages of naloxone outreach and distribution efforts. In addition, our findings are specific to the US and may not be generalizable to other countries. Fifth, the elapsed time of two years between the qualitative and survey data collections may be viewed as a limitation. During those two years changing social environmental factors, including COVID-19, may or may not have impacted naloxone perceptions. Finally, the relationships between bystander participants and PWUIO also differed slightly between qualitative and quantitative data collections. However, we found no significant differences when comparing primary survey outcomes as a function of bystander-PWUIO relationships, suggesting that these differences likely did not bias outcomes but instead, provided a more comprehensive view of bystander experiences.

## Conclusions

While naloxone is highly available under Massachusetts’ NSO program [16], many potential overdose “bystanders” do not utilize pharmacies as an access point for naloxone acquisition. Misconceptions about opioid overdose risk and naloxone, as well as stigmatization of PWUIO and secondary stigma experienced by bystanders, serve as barriers to naloxone uptake. Mutual support groups were critical in decreasing psychosocial barriers and provided a free source of naloxone and overdose response training. Incorporating the social processes and logistical support provided in mutual support groups into pharmacy and healthcare practices has the potential to address barriers to risk assessment and increase naloxone uptake among potential opioid overdose bystanders. For those without access to naloxone through support groups, reclassifying naloxone as an over-the-counter medication is one intervention that may increase pharmacy-based naloxone uptake by potential opioid overdose bystanders.

## Data Availability

Due to ethical restrictions, datasets generated and analyzed during the current study are not publicly available as they contain potentially identifying information of a sensitive nature. Reasonable requests for access should be directed to the corresponding author.

## Abbreviations

NSO: Naloxone standing order
SSP: Syringe services program
PWUIO: People who use illicit opioids
SUD: Substance use disorder
OUD: Opioid use disorder
